# Exploring the association between phytopharmaceutical use and antibiotic prescriptions in upper respiratory infections: results from a German cohort study evaluating the impact of naturopathy qualifications of general practitioners using routine data

**DOI:** 10.3389/fmed.2024.1440632

**Published:** 2024-10-18

**Authors:** Anna-Jasmin Wetzel, Gunter Laux, Stefanie Joos, Berthold Musselmann, Jan Valentini

**Affiliations:** ^1^Institute of General Practice and Interprofessional Care, Tübingen University Hospital, Tübingen, Germany; ^2^Department of General Medicine and Health Service, Heidelberg University Hospital, Heidelberg, Germany; ^3^Practice Dr. Musselmann, Wiesloch, Germany

**Keywords:** antibiotic resistance, phytopharmaceuticals, phytotherapeutica, complementary and integrative medicine, naturopathy, upper respiratory infections, cohort study, primary care

## Abstract

**Background:**

Antibiotic resistance is a significant global health threat, exacerbated by inappropriate prescribing practices, particularly for upper respiratory infections that are predominantly viral. Complementary and Integrative Medicine (CIM), including the use of phytopharmaceuticals, offers a potential strategy to reduce antibiotic prescriptions.

**Objective:**

This study aimed to describe the impact of General Practitioners’ (GPs) naturopathy (NP) qualifications and phytopharmaceutical prescriptions on the rate of antibiotic prescribing for upper respiratory infections (RTI).

**Methods:**

We conducted a retrospective cohort study using routine data from the CONTinuous morbidity registration Epidemiologic NeTwork (CONTENT), which includes over 200,000 patients across four federal states in Germany. The study included data from *n* = 36 GPs who recorded at least one ICD-10 diagnosis of RTI. Antibiotic and phytopharmaceutical prescriptions were identified and analyzed through mixed-effects logistic regression models to explore the influence of GPs’ naturopathy qualifications and phytopharmaceutical use on antibiotic prescribing patterns.

**Results:**

The study included 40,344 patients managed by 36 GPs. Prescriptions of phytopharmaceuticals significantly reduced the likelihood of antibiotic use (OR 0.48, 95% CI 0.45–0.52). Additionally, holding a naturopathy qualification was associated with lower rates of antibiotic prescriptions (OR 0.73, 95% CI 0.69–0.78). The interaction between naturopathy qualification and phytopharmaceutical prescriptions also showed a significant effect (OR 1.43, 95% CI 1.27–1.62). Patient’s year of birth influenced prescribing patterns indicating a reduction of antibiotic prescriptions for younger patients, while patients’ gender did not reveal a significant effect.

**Conclusion:**

Prescriptions of phytopharmaceuticals were significantly associated with a decrease antibiotic prescriptions among GPs, especially when combined with naturopathy qualifications. Training in naturopathic approaches could enhance antibiotic stewardship efforts in primary care settings, suggesting that broader integration of CIM elements into medical training could be beneficial in mitigating antibiotic resistance.

## 1 Introduction

Antibiotic resistance has emerged as a significant and escalating concern in recent years. The World Health Organization declared antibiotic resistance in the Global antimicrobial resistance (AMR) and use report of 2021 as one of the top 10 global public health threats facing humanity (1). AMR develops not only in the context of human medicine but also as a result of antimicrobial consumption in animals, which has enabled large-scale breeding for meat production (2). In 2019, almost 1.3 million deaths worldwide were attributed to multi-resistant pathogens (3), which alone cost the health systems in the EU/EEA an additional approximately 1.1 billion euros per year (4). In Europe alone, approximately 33,000 deaths occur annually as a result of infections caused by antibiotic-resistant bacteria (5). Alarmingly, up to 50% of antibiotic prescriptions in humans are estimated to be not indicated (6). According to the German Antibiotic Resistance Strategy 2030 (DART 2030), about 85% of antibiotics are prescribed in outpatient settings, underscoring the importance of reinforcing appropriate antibiotic usage (7). Antibiotic Stewardship (ABS) is emphasized as an essential strategy for optimizing antibiotic use and mitigating the escalating challenge of antimicrobial resistance according to DART 2023 (7). By focusing on precise diagnostics, timely and appropriate antibiotic selection, and clear guidance on treatment specifics, ABS contributes significantly to responsible antibiotic management in both inpatient and outpatient settings (7). Emphasizing education and behavior reflection among healthcare professionals, including physicians, dentists, and veterinarians, is key to ingraining responsible antibiotic practices (7).

In cases of acute upper RTI, antibiotics are prescribed in 25–41% of instances, despite the majority of these infections being caused by viral pathogens (8, 9). Consequently, antibiotic prescriptions often are inappropriate and ineffective. Further, only half of these prescriptions align with clinical recommendations (10). Notably, general practitioners account for 85% of antibiotic prescriptions (7), which is unsurprising given that acute lower and upper RTI rank among the most common reasons for visits to general practices (11). Consequently, general practitioners represent a crucial starting point for potentially reducing overall antibiotic usage as well as reducing non-indicated antibiotic prescriptions and mitigating associated issues such as antibiotic resistance (12).

One approach to reducing unnecessary antibiotic prescriptions could involve strategies from Complementary and Integrative Medicine (CIM) (13), particularly the use of phytopharmaceuticals, which have been associated with a decrease in antibiotic use (14). Phytopharmaceuticals are defined as medicinal products derived from plant materials (including extracts, tinctures, and compounds) used for therapeutic purposes. Phytopharmaceuticals, unlike traditional herbal remedies such as teas or decoctions, are typically standardized to ensure consistent potency and efficacy (15, 16). When treating acute upper RTI, phytopharmacological substances have undergone extensive investigation in experimental studies, with numerous compounds demonstrating efficacy (17–23). Additionally, patients treated with phytopharmaceuticals experienced a significantly reduced risk of prolonged sick leave (14). In a retrospective cohort study by Martin et al. Pelargonium sidoides root and thyme extract were found to be particularly effective in adult GP patients, while pediatric patients benefited most from Pelargonium sidoides root extract in combination with thyme and ivy extract, as well as thyme and primrose root extract (14).

Despite patients often expecting antibiotics prescriptions (24), phytopharmaceuticals may offer in many cases a preferable alternative that could potentially contribute to reducing antibiotic-resistant bacteria and associated infections. GPs with additional qualifications in naturopathy are known to prescribe more phytopharmaceuticals in general compared to GPs without such qualifications (25). The term “naturopathy” has different meanings in various contexts and countries. For the purposes of this publication, naturopathy refers to the additional qualification in CIM for physicians awarded by the German Medical Association. Upon completing their postgraduate education, physicians in Germany have the opportunity to pursue structured additional qualifications in the field of CIM, which are conferred by the medical association (26). These include Acupuncture, Homeopathy, Manual Medicine/Chirotherapy, Medical Balneology and Climatology, Naturopathy, and Physical Therapy (26). Furthermore, it is important to note that obtaining an additional qualification in Naturopathy (NP) requires 3 months of training under a certified physician, 80 h of case-seminar supervision, and 160 h in a seminar program, totaling 240 h of advanced education (26). This training encompasses various facets of naturopathy including phytotherapy, balneotherapy, massage, manual diagnostics, nutritional medicine, regulative therapy, physical therapy, and neural therapy. Notably, phytotherapy (including phytopharmaceuticals) is a key component among these NP approaches, underscoring the significance of herbal treatments in this field of medicine (26).

Thus, the objective of this study is to examine whether GPs holding an additional qualification in naturopathy prescribe fewer antibiotics for acute upper RTI compared to those without this qualification. Additionally, we aim to assess which antibiotics and/or phytopharmaceuticals are commonly prescribed for upper RTI and whether phytopharmaceutical usage correlates with reduced antibiotic prescriptions.

## 2 Materials and methods

This study is a retrospective cohort analysis using routine data from general practices within the German healthcare system. The STROSA 2 checklist was applied to this manuscript (27).

### 2.1 Data source

Data were obtained from the CONTinuous morbidity registration Epidemiologic NeTwork (CONTENT), a comprehensive general practice research network in Germany (28). CONTENT facilitates the continuous, episodic recording of primary care data, incorporating information from over 200,000 patients and upwards of 4 million patient encounters. The network spans rural, suburban, and urban areas across four federal states in Germany: Baden-Württemberg, Bavaria, Lower Saxony, and Rhineland-Palatinate. The dataset comprises routine claims data from General Practitioners (GPs) as typically gathered within the German healthcare framework. The CONTENT Register was developed and put into operation by the Department of General Medicine and Health Services Research at the University Hospital Heidelberg.

For this study, the CONTENT research network was expanded between April 2009 and March 2015 to include 11 GP practices with additional naturopathic qualifications. This meant that a total of 41 GP practices, 11 of which had additional naturopathic qualifications, were included in the study period. As phytopharmaceuticals are over-the-counter medications that are often recommended but not formally prescribed by GPs, GPs were specifically instructed prior to the start of the CONTENT registry to prescribe each phytopharmaceutical on a prescription (and not just give a verbal recommendation, as is often done in clinical practice) to ensure that the data also accurately reflected the prescribing of phytopharmaceuticals (25).

### 2.2 Legal basis

The data provision took place before the EU-GDPR. At the time of data collection, the medical professional code of Baden-Württemberg (§15, Para. 3) applied: As a basic principle, only anonymized data are transmitted. For each patient, the CONTENT EPR contains a case number, the patient’s year of birth, and the patient’s gender but not the name or address. Thus, it is not possible to determine a patient’s identity, and the implementation of extensive data security mechanisms is not needed. Moreover, the German Data Protection Act allows the transmission of anonymized patient data for scientific purposes without the explicit permission of patients.

The Ethics Committee of the University Hospital Heidelberg has dealt extensively with the CONTENT project concerning ethical and data protection aspects and, after a consenting assessment, issued a positive vote (442/2005).

### 2.3 Data protection

The data processing of the Department of General Medicine and Health Services Research at the University Hospital Heidelberg is carried out with systems that are approved, implemented, maintained, and secured by the Center for Digitalization and Information Technology (ZDI) of the University Hospital Heidelberg. The ZDI is a certified institution that either carries out the necessary technical and organizational measures for data processing in accordance with the EU General Data Protection Regulation itself or enables these measures.

### 2.4 Data flow

Encrypted exports (https) were initiated quarterly by the general practitioner from each individual practice. The quarterly exports were received on a central server behind a firewall. These data were then imported quarterly into the central CONTENT database, which is inaccessible from the outside.

### 2.5 Eligibility criteria

The study included data from GPs contributing their routine data to CONTENT between January 1, 2010, and December 31, 2014. This encompassed data on 53,572 patients who had at least one ICD-10 diagnosis starting with “J.” Diagnoses related to upper RTI, as per the ICD-10 codes, were selected and are detailed in Appendix 1. Exclusions were made for diagnoses where antibiotic prescriptions are generally not indicated (e.g., J09) or in cases of chronic conditions such as COPD. The study required the join of diagnosis and prescription data, both of which were derived from secondary sources.

Initially, all antibiotic prescriptions were identified using the Anatomical Therapeutic Chemical (ATC) classification system. Subsequently, two physicians evaluated these prescriptions to determine their appropriateness for the use in upper RTI, excluding any that were deemed not suitable (e.g., Antibiotics that are either not approved for treating respiratory infections, not recommended by guidelines, or exclusively administered intravenously). Phytopharmaceuticals were also classified using the ATC Code, specifically searching for the term “pflanzl” within the chemical substance subgroup. Following this step, two physicians reviewed the phytopharmaceuticals identified, selecting those appropriate for treating upper RTI according to according to the currently valid clinical guidelines. Prescriptions were considered relevant if prescribed within a maximum of 14 days following the diagnosis.

### 2.6 Outcome and predictor variables

The study’s primary outcomes were antibiotic prescriptions for an ICD-10 J-Diagnosis. The outcome was binary coded (0/1). Predictor variables included the additional qualifications of general practitioners (GPs), specifically their certification in naturopathy and phytopharmaceutical prescriptions. Patient’s year of birth (YOB) and patient’s gender were also considered as predictor variables. YOB was included as a continuous variable and was scaled to address convergence issues to ensure the identifiability and proper calculation of the general linear mixed model. The unit for YOB was expressed as the standard deviation (SD) of the overall age distribution.

### 2.7 Data preprocessing and statistical analysis

Data preprocessing and analysis were performed using R version 4.3.2 (29), utilizing the tidyverse package (30) for data preprocessing and the gtsummary (31) package for generating descriptive tables.

Mixed-effects logistic regression models were constructed, building upon simpler models. These included a model with only an intercept, a model incorporating patient’s year of birth (YOB) and patient’s gender as covariates, a model integrating the aforementioned predictors alongside the additional NP qualification and phytopharmaceutical prescriptions, and a full model encompassing all explanatory variables along with the interaction of phytopharmaceutical prescriptions and the additional qualification in naturopathy. A random effect for patients was introduced to address data clustering (multiple observations per patient). The analysis was conducted using the R package glmmtmb (32), with result tables produced utilizing the sjPlot (33) package. Overdispersion statistics were derived with the DHARMa (34) package.

## 3 Results

### 3.1 Sample description

The cohort comprised 36 GPs, with 11 (27%) possessing additional qualifications in NP and 25 (73%) without the additional NP qualification. Applying the eligibility criteria, resulted in *N* = 40,344 Patients and *N* = 81,057 diagnoses included. An overview of patient demographic can be found in Table 1. Patients year of birth ranged from 1903 to 2014 and 57.2% of the participants were female. The most frequently prescribed phytopharmaceuticals and antibiotics can be found in Tables 2, 3. An overview of the 10 most coded ICD-10 diagnosis can be found in Table 4.

**Table 1:** Sociodemographic characteristics of the patients.

Patient demographics	No NP qualification, *N* = 29,947^1^	NP qualification, *N* = 10,397^1^	Total *N* = 40,344^1^
Year of birth	1972 (20.2)	1973 (19.8)	1972 (20.1)
Gender (female)	17,061 (57.0)	6,028 (58.0)	23,089 (57.2)
Type of health insurance			
Satutory	26,991 (90.1%)	9,603 (92.4)	36,594 (90.7)
Private	2,956 (9.9%)	794 (7.6%)	3,750 (9.3)

1 Mean (SD); n (%).

**Table 2:** Ten most frequently prescribed phytopharmaceuticals.

10 most frequently prescribed phytopharmaceuticals	*N* = 19,317^1^
Sinupret^®^	5,358 (27.7)
Gelomyrtol^®^	3,491 (18.1)
Bronchipret^®^	1991 (10.3)
Prospan^®^	1888 (9.8)
Bronchicum^®^	1794 (9.3)
Umckaloabo^®^	1,529 (7.9)
Bronchoforton^®^	1,049 (5.4)
Muc Sabona^®^	789 (4.1)
Angocin^®^	714 (3.7)
Imupret^®^	714 (3.7)

1 n (%).

**Table 3:** Ten most frequently prescribed antibiotics.

10 most frequently prescribed antibiotics	*N* = 56,102^1^
Amoxicillin with or without Beta-Lactase-Inhibitors	11,127 (19.8)
Clarithromycin	8,794 (15.7)
Cefuroxime	7,844 (14.0)
Ciprofloxacin	5,666 (10.1)
Cefpodoxime	5,331 (9.5)
Azithromycin	5,062 (9.0)
Levofloxacin	4,114 (7.3)
Phenoxymethylpenicillin	3,186 (5.7)
Doxycycline	2,604 (4.6)
Roxithromycin	2,374 (4.2)

1 n (%).

**Table 4:** Ten most frequently coded ICD-10 diagnosis, with the frequency and percentages of prescribed antibiotics and phytopharmaceuticals.

10 most frequently coded ICD-10 Diagnosis		No NP qualification	NP qualification	*N* = 77,610
J06.9	Acute upper respiratory infection, unspecified	28,089 (48.3)	9,951 (43.5)	38,040 (49.0)
	Prescribed antibiotics	3,925 (14)	2,684 (27)	
	Prescribed phytopharmaceuticals	5,842 (21)	1,758 (18)	
J02.9	Acute pharyngitis, unspecified	11,961 (20.6)	3,032 (13.3)	14,993 (19.3)
	Prescribed antibiotics	8,201 (69)	1,116 (37)	
	Prescribed phytopharmaceuticals	869 (7.3)	381 (13)	
J20.9	Acute bronchitis, unspecified	7,868 (13.5)	2,335 (10.2)	10,203 (13.1)
	Prescribed antibiotics	4,058 (52)	1,053 (45)	
	Prescribed phytopharmaceuticals	1,175 (15)	476 (20)	
J03.9	Acute tonsillitis, unspecified	4,504 (7.8)	1,340 (5.9)	5,844 (7.5)
	Prescribed antibiotics	3,359 (75.0)	1,005 (75.0)	
	Prescribed phytopharmaceuticals	156 (3.5)	117 (8.7)	
J04.0	Acute laryngitis	1,609 (2.8)	882 (3.9)	2,491 (3.2)
	Prescribed antibiotics	413 (26)	278 (32)	
	Prescribed phytopharmaceuticals	110 (6.8)	145 (16)	
J06.0	Acute laryngopharyngitis	1,564 (2.7)	68 (0.3)	1,632 (2.1)
	Prescribed antibiotics	808 (52.0)	29 (43.0)	
	Prescribed phytopharmaceuticals	112 (7.2)	16 (24.0)	
J06.8	Other acute upper respiratory infections of multiple sites	770 (1.3)	602 (2.6)	1,372 (1.8)
	Prescribed antibiotics	355 (46.0)	103 (17.0)	
	Prescribed phytopharmaceuticals	116 (15.0)	150 (25.0)	
J04.2	Acute laryngotracheitis	573 (1.0)	480 (2.1)	1,053 (1.4)
	Prescribed antibiotics	323 (56.0)	136 (28.0)	
	Prescribed phytopharmaceuticals	110 (19.0)	72 (15.0)	
J01.1	Acute frontal sinusitis, unspecified	93 (0.2)	927 (4.0)	1,020 (1.3)
	Prescribed antibiotics	37 (40.0)	228 (25.0)	
	Prescribed phytopharmaceuticals	40 (43.0)	259 (28.0)	
J01.9	Acute sinusitis, unspecified	701 (1.2)	261 (1.1)	962 (1.2)
	Prescribed antibiotics	438 (62.0)	104 (40.0)	
	Prescribed phytopharmaceuticals	185 (26.0)	86 (33.0)	

### 3.2 Generalized linear mixed model

#### 3.2.1 Summary results

Overdispersion statistics can be found in Appendix 2. They were visually checked as significance is a known problem considering the large numbers of measurements. There were no relevant discrepancies discovered. The variance of the random effect ranged between 1.82 and 1.76, the random effect was statistically significant further indicating underlying heterogeneity between the individuals (Table 5). The Model with the best Akaike information criterion (AIC) (101290) was Model 4; including all predictors as well as the interaction term of the NP qualification and phytopharmaceutical prescriptions. An overview of all models and predictors can be found in Table 5.

**Table 5:** Model parameters for Models 0–4.

	Intercept-only model			Model 1			Model 2			Model 3			Model 4		
Predictors	Odds ratios	CI	*p*	Odds ratios	CI	*p*	Odds ratios	CI	*p*	Odds ratios	CI	*p*	Odds ratios	CI	*p*
Intercept	0.47	0.46–0.49	<0.001	0.46	0.44–0.48	<0.001	0.49	0.47–0.51	<0.001	0.54	0.52–0.56	<0.001	0.55	0.53–0.57	<0.001
Gender (female)				1.03	0.98–1.08	0.205	1.03	0.99–1.08	0.146	1.03	0.99–1.08	0.150	1.04	0.99–1.08	0.141
Year of birth scaled				0.82	0.80–0.84	<0.001	0.82	0.80–0.84	<0.001	0.82	0.80–0.84	<0.001	0.82	0.80–0.84	<0.001
NP qualification (yes)							0.76	0.72–0.80	<0.001	0.78	0.74–0.82	<0.001	0.73	0.69–0.78	<0.001
Phytopharmaceutical (yes)										0.53	0.50–0.56	<0.001	0.48	0.45–0.51	<0.001
Interaction phytopharmaceutical (yes) and NP qualification (yes)													1.43	1.27–1.62	<0.001
Random effects															
σ^2^	3.29			3.29			3.29			3.29			3.29		
τ_00_	1.82 _cid_			1.80 _cid_			1.80 _cid_			1.76 _cid_			1.76 _cid_		
ICC	0.36			0.35			0.35			0.35			0.35		
N	40,344 _cid_			40,344 _cid_			40,344 _cid_			40,344 _cid_			40,344 _cid_		
Observations	81,057			81,057			81,057			81,057			81,057		
AIC	102239.572			101941.078			101841.618			101321.117			101290.370		

#### 3.2.2 Patient’s year of birth and patient’s gender

Patient’s year of birth (YOB) was consistently identified as a significant predictor across all models, with odds ratios (ORs) remaining stable at 0.82 (95% CI: 0.80–0.84). Accordingly, antibiotic prescriptions decreased with an odds ratio (OR) of 0.82 for each increase in the scaled variable “year of birth” (where one unit equals 20.1 years), suggesting that younger individuals were less likely to receive antibiotics. Patient’s gender, however, did not show a significant impact in Models 0–4, with ORs close to 1 across all models (OR: 1.03–1.04; 95% CI: 0.99–1.08).

#### 3.2.3 NP qualification

The inclusion of additional qualifications in NP as a variable revealed a notable impact across all models where it was included. ORs ranged from 0.76 to 0.73 (95% CI: 0.69–0.82), indicating a reduction on antibiotic prescription rates.

#### 3.2.4 Phytopharmaceutical prescriptions

Phytopharmaceutical prescriptions emerged as another significant predictor, with ORs between 0.53 and 0.48 (95% CI: 0.45–0.56), pointing to a reduction on antibiotic prescriptions.

#### 3.2.5 Interaction: phytopharmaceutical prescriptions and NP qualification

**Figure 1:**
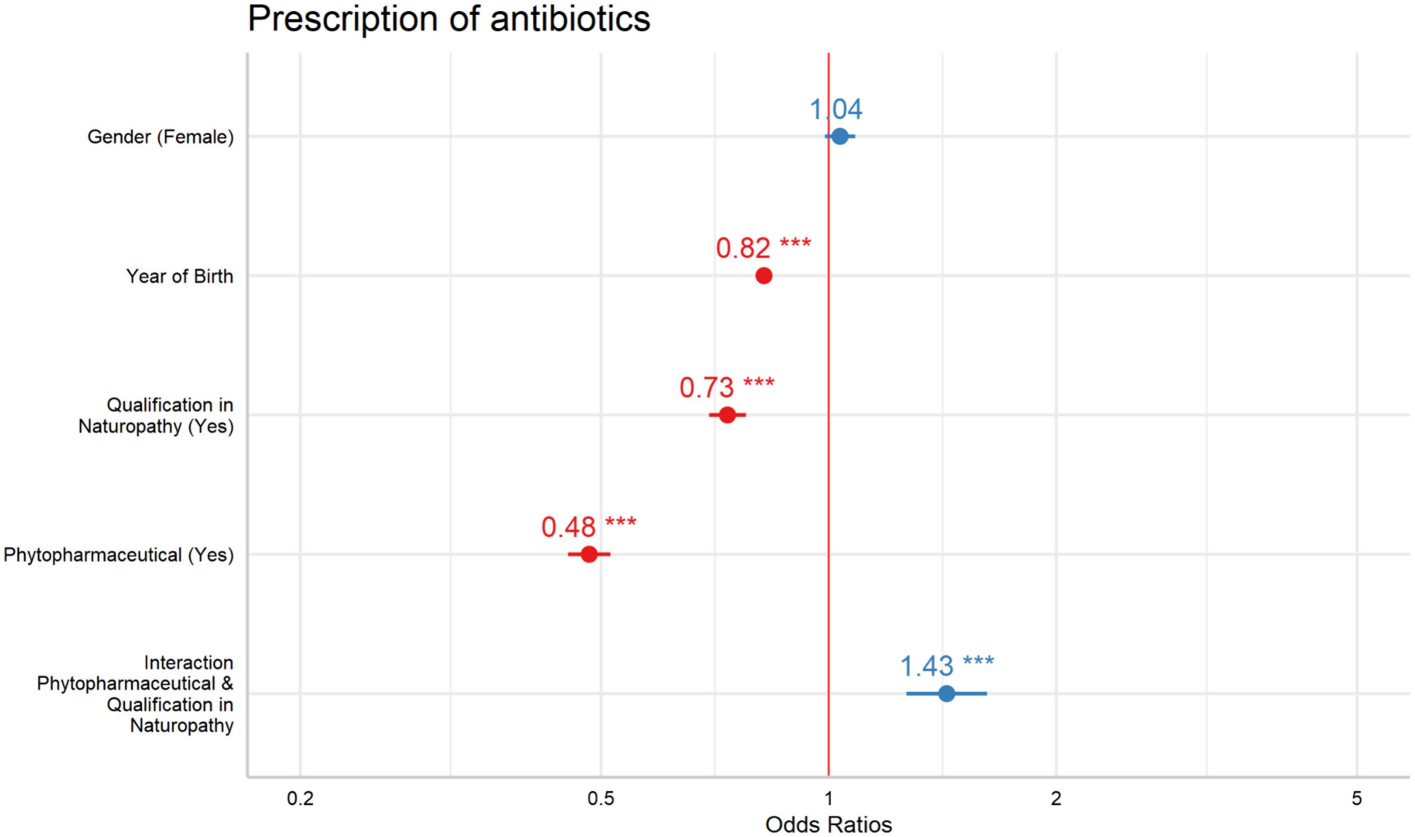
Odds ratios and confidence intervals for model 4.

The interaction between phytopharmaceutical prescriptions and an additional NP showed a significant effect, leading to a stratified analysis based on the presence of naturopathy qualifications (Table 6). For GPs with NP qualifications, the OR for phytopharmaceutical prescriptions was 0.69 (95% CI: 0.62–0.76). For GPs without an additional NP qualification, the OR was 0.48 (95% CI: 0.45–0.52). Both predictors revealed a significant effect within their respective models (Figure 1).

**Table 6:** Stratified *post hoc* analysis for NP qualification.

	Model NP qualification			Model no NP qualification		
Predictors	Odds ratios	CI	*p*	Odds ratios	CI	*p*
Intercept	0.35	0.33–0.39	<0.001	0.57	0.54–0.59	<0.001
Gender (female)	1.21	1.10–1.33	<0.001	0.98	0.93–1.04	0.566
Year of birth scaled	0.83	0.79–0.87	<0.001	0.82	0.80–0.84	<0.001
Phytopharmaceutical prescription	0.69	0.62–0.76	<0.001	0.48	0.45–0.52	<0.001
Random effects						
σ^2^	3.29			3.29		
τ_00_	2.02 _cid_			1.69 _cid_		
ICC	0.38			0.34		
N	10,397 _cid_			29,947 _cid_		
Observations	20,590			60,467		
AIC	25171.864			76104.223		

#### 3.2.6 *Post hoc* analysis: differences in prescriptions stratified for NP qualification

To further investigate the effect of the identified significant interaction of NP qualification and phytopharmaceutical prescriptions a *post hoc* analysis was conducted. Hereby the primary outcome was re-structured to also describe simultaneous prescriptions of antibiotics and phytopharmaceuticals. A Pearson’s Chi Square test revealed differences in the prescription patterns between GPs with and without NP qualification considering prescriptions of antibiotics, phytopharmaceuticals, both types of medication or no prescriptions. GPs with a qualification in NP prescribed less antibiotics, more phytopharmaceuticals, more frequently a combination of both types of medication and more frequently no medication compared to GPs without a NP qualification (Table 7).

**Table 7:** *Post hoc* analysis—prescriptions stratified for NP qualification.

Qualification in naturopathy	No, *N* = 60,467*^1^*	Yes, *N* = 20,590^1^	*p*-value^2^
Prescriptions			<0.001
Antibiotics	21,161 (35%)	6,090 (30%)	
Phytopharmaceutical	6,557 (11%)	2,573 (12%)	
Both	2,427 (4.0%)	1,018 (4.9%)	
Nothing	30,322 (50%)	10,909 (53%)	

1 n (%).

2 Pearson’s Chi-squared test.

## 4 Discussion

### 4.1 Main results

The recent study explored how the variables—patient’s year of birth, patient’s gender, phytopharmaceutical prescriptions, NP qualification, and the combined impact of phytopharmaceutical prescriptions and NP qualification—affect antibiotic prescriptions for upper RTI. As expected all predictors besides patient’s gender significantly influenced antibiotic prescription rates. Phytopharmaceutical prescriptions led to the most substantial decrease in antibiotic use, followed by having a qualification in NP and then by patient’s year of birth. The concurrent prescription of phytopharmaceuticals along with holding a NP qualification had a notable impact, leading to a reduction in antibiotic prescriptions among GPs possessing an NP qualification while simultaneously prescribing phytopharmaceuticals. Surprisingly, GPs without additional qualification in NP who prescribed phytopharmaceuticals, prescribed even less antibiotics.

In the subsequent paragraph the influence of the significant predictors on antibiotic prescriptions will be discussed.

### 4.2 Patient’s year of birth and patient’s gender

While year of birth was consistently identified as a predictor, with older patients likely receiving more antibiotics, patient’s gender did not significantly influence prescription patterns. The age-related trends might be attributed to a higher co-morbidities and frailty and is in line with other recent findings (35). However, the lack of significant impact of patient’s gender suggests that prescriptive decisions are more strongly influenced by clinical factors and individual qualifications rather than patient gender (36).

### 4.3 Phytopharmaceutical prescription

Consistent with findings from other studies (12), phytopharmaceutical prescriptions have been found to significantly reduce antibiotic use in treating upper RTI. The effects demonstrated in recent literature also seem applicable to the context of GPs in Germany, suggesting a broader relevance and potential for phytopharmaceuticals in clinical practice consisting of two steps. Firstly, unnecessary antibiotic prescriptions, often administered for upper RTIs which are predominantly viral (37), are not indicated. This action can reduce adverse effects for patients and help mitigate the development of antibiotic resistance. Secondly, evidence-based phytopharmaceuticals should be more widely utilized to alleviate symptoms. Additional research already highlights the effectiveness of phytopharmaceuticals (17–23). In light of our data, it appears that phytopharmaceuticals contribute to the decrease in antibiotic prescriptions for upper RTI. Additional training for physicians in outpatient settings on phytopharmaceuticals could further help reduce unnecessary antibiotic prescriptions.

### 4.4 NP qualification

To our knowledge, this is the first study to investigate the impact of additional qualifications in NP on antibiotic prescriptions in upper RTI. As anticipated, possessing an additional certification in NP was associated with reduced antibiotic prescriptions in general practice settings in Germany. These GPs’ broader training and awareness in complementary and integrative medicine, including phytotherapy as part of their NP qualification, likely predisposes them to favor non-antibiotic treatments. Similar results were found by van der Werf et al. for NHS England GP practices employing GPs with additional CIM training had lower antibiotic prescribing rates for RTI compared with GPs without additional CIM training. In contrast to this study, we included GPs with a specific qualification in naturopathy, whereas van der Werf et al. included GPs with a wider range of different specializations in CIM (acupuncture, anthroposophic medicine, homeopathy), making the results and implications somewhat difficult to compare, as CIM GPs may have adopted different strategies to decrease antibotic prescribing (38). Nevertheless, both of these findings emphasize the potential benefits of incorporating naturopathic (and/or CIM) training or modules into medical education to enhance multiple therapeutic options for their own prescribing practice. This finding therefore highlights the potential benefits of further training in CIM, suggesting that early integration of CIM-specific competencies in undergraduate as well as in postgraduate training could be particularly beneficial for GPs (39, 40). Valentini et al. (40) identified 16 competencies for postgraduate training of general practitioners (GPs) within the German healthcare system. Among these, competency 7 emphasizes the ability to use common phytotherapeutics and supplements for frequent consultation issues, such as pain, fever, and uncomplicated infections, thus providing a suitable framework for additional training.

### 4.5 Interaction of phytopharmaceutical prescriptions and NP qualification

The observed outcome of combining an additional NP qualification with the prescription of phytopharmaceuticals did not align with our expectations. Surprisingly, GPs lacking an additional NP title showed a greater reduction in antibiotic prescriptions when they also prescribed phytopharmaceuticals.

This discrepancy may arise from GPs with an NP qualification tending to co-prescribe antibiotics and phytopharmaceuticals more frequently than GPs without an additional NP qualification. In contrast, GPs without an additional NP qualification seem to prefer an “either/or” approach, choosing more often to prescribe either antibiotics alone or substitute them with phytopharmaceuticals, rather than combining the two. To explore this hypothesis further, a stratified *post hoc* analysis was conducted. The analysis revealed that GPs with NP qualifications co-prescribed both types of medication in 5% of cases, compared to 4% for GPs without the qualification, underscore this hypothesis. Another reason that may explain the interaction between phytopharmaceutical prescriptions and the additional qualification is that prescribing antibiotics and phytopharmaceuticals is a two-step process. First, non-indicated antibiotic prescriptions must be reduced. In the second step, phytopharmaceutical prescriptions should be increased when indicated to help with symptom relief.

Additionally, we could not account for the use of home remedies, such as inhalation or rinses with, e.g., sodium chloride solutions, which may also have an impact on reducing symptoms. In addition to home remedies, factors such as professional networks and collaborations with laboratories routinely providing information on local resistance data are recognized for their role in shaping antibiotic prescribing behaviors. These elements might also affect how phytopharmaceutical prescriptions and naturopathic practitioner qualifications interact, varying with regional conditions (41). Additional evidence from qualitative studies investigating these different aspects, as well as the motives for antibiotic and phytopharmaceutical prescriptions, are necessary to understand this interaction in depth.

### 4.6 Clinical and policy implications

These findings have significant implications for clinical practice and health policy. Encouraging the integration of NP training and the use of phytopharmaceuticals could be a part of a feasible strategy to reduce antibiotic prescriptions, particularly in the context of upper RTI where overprescription is common. In Germany, some phytopharmaceuticals are already included in clinical guidelines for GPs, suggesting their broader implementation in treating upper RTIs as an evidence-based strategy (42, 43). Additionally, our research supports ongoing education for healthcare professionals on the risks of antibiotic resistance and the benefits of complementary therapies. The over-prescription of antibiotics, often driven by perceived patient pressure and the fear of disease complications as noted by Altiner et al. (12) highlights the need for improved patient education regarding upper RTIs (44). Home remedies, for instance, could serve as effective tools for patients managing self-limiting conditions. According to a 2014 study, a significant number of patients use home remedies to manage symptoms of colds, with 97% reporting improved well-being and reduced symptoms (45). The most commonly used home remedies include hot steam inhalation, hot lemon drinks, and honey (45). These remedies also play a role in patients’ own symptom management for minor health complaints, indicating that those who use home remedies take a more active interest and role in their health (45). GPs should promote the use of home remedies for managing symptoms. A comprehensive study from Germany revealed promising outcomes and produced informational materials that were well-received by both doctors and patients. Information leaflets offer an accessible approach that can help align patient expectations about medication use and increase self-efficacy, while also enhancing the visibility and use of home remedies for symptom relief (46).

Health policymakers should consider incentivizing the reimbursement for evidence-based phytopharmaceuticals for specific indications like RTIs. Currently, patients in Germany must pay for these treatments out-of-pocket, whereas antibiotic treatments are covered by statutory health insurance. Addressing this disparity could help reduce unnecessary antibiotic prescriptions.

### 4.7 Strengths, limitations, and future research

A major strength of this study is its large patient sample size and the utilization of data from general practice settings, which enhances the applicability of the findings. An additional strength of this study lies in the systematic documentation of phytopharmaceuticals facilitated by the structured training of GPs. Phytopharmaceuticals are often recommended by physicians without formal prescriptions, so patients typically acquire them over-the-counter based on this advice, a practice not reflected in existing routine datasets.

The data analyzed in this study comprehensively represents the full spectrum of prescribed phytopharmaceuticals. However, the study has several limitations. It focuses generally on antibiotic prescriptions without determining whether they were indicated or not, as it was not possible to assess the adequacy of antibiotic prescriptions with the presented data. However, the study is subject to several limitations. It primarily examines antibiotic prescriptions without determining whether they were warranted, as the data provided did not allow for an assessment of the appropriateness of these prescriptions. Nonetheless, other studies have suggested that upper respiratory tract infections (RTIs) are seldom caused by bacterial pathogens. For instance, a 2019 study investigating upper RTIs found that only 11.6% of cases were attributable solely to bacterial infections (37). Additionally, the analysis included only a limited set of covariates and did not account for comorbidities. Although prior analyses suggest similar morbidity levels across practices (25), the sample may not be fully representative of the entire German population. In addition, the data presented is from the period 2010–2014 and could not be updated due to limited access to more recent records; however, it is unlikely that prescribing practices have changed significantly so it can be assumed that the results are still valid today. The retrospective design and reliance on routine data further necessitate a cautious interpretation of the results. Future research should aim to prospectively validate these findings and explore how naturopathy training specifically influences prescription behaviors. Expanding the study to include diverse geographic regions and healthcare systems would also improve the generalizability of the results.

## 5 Conclusion

This study illustrates the beneficial role of phytopharmaceuticals and naturopathy qualifications in reducing antibiotic prescriptions among GPs in upper RTI. It highlights the importance of complementary approaches and specialized training in fighting the global challenge of antibiotic resistance. Encouraging broader adoption of these practices could significantly contribute to more sustainable healthcare practices and better patient outcomes in the face of escalating antimicrobial resistance.

## Data Availability

The raw data supporting the conclusions of this article will be made available by the author on reasonable request in anonymised form in accordance with the institutional regulations and the General Data Protection Regulation.
