# Supplementary figures and images for: Exploring the association between phytopharmaceutical use and antibiotic prescriptions in upper respiratory infections: results from a German cohort study evaluating the impact of naturopathy qualifications of general practitioners using routine data
Source: Front Med (Lausanne). 2024 Oct 18;11:1440632. doi: 10.3389/fmed.2024.1440632 (PMC11527615; doi:10.3389/fmed.2024.1440632)

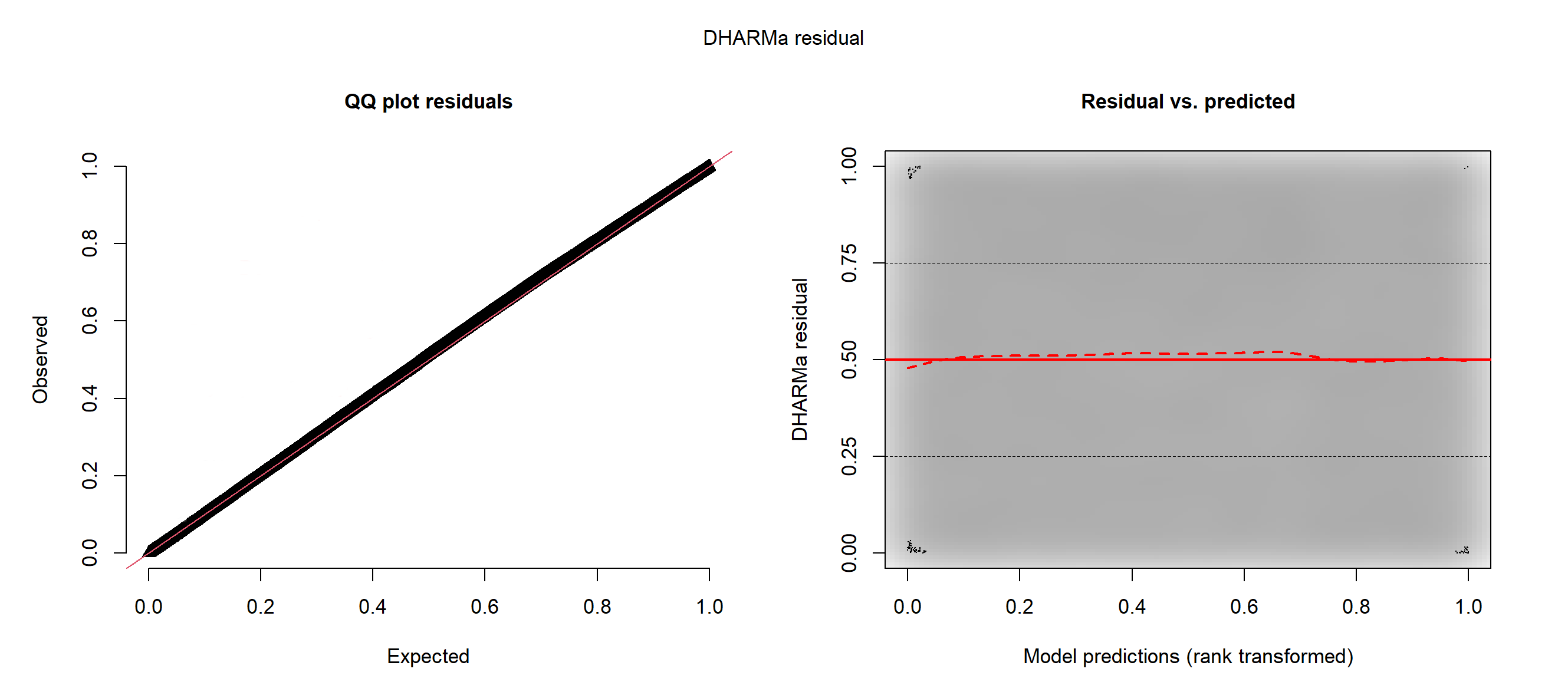

Supplement: Supplementary file 1 [file Image_1.tif]
