# Supplementary material for: Exploring the association between phytopharmaceutical use and antibiotic prescriptions in upper respiratory infections: results from a German cohort study evaluating the impact of naturopathy qualifications of general practitioners using routine data
Source: Front Med (Lausanne). 2024 Oct 18;11:1440632. doi: 10.3389/fmed.2024.1440632 (PMC11527615; doi:10.3389/fmed.2024.1440632)
Supplement: Supplementary file 2 [file Table_1.docx]

|  | |  |  |
| --- | --- | --- | --- |
| *10 most frequently coded ICD-10 Diagnosis* | | | *Count* |
| J06.9 | Acute upper respiratory infection, unspecified | | 38040 |
| J02.9 | Acute pharyngitis, unspecified | | 14993 |
| J20.9 | Acute bronchitis, unspecified | | 10203 |
| J03.9 | Acute tonsillitis, unspecified | | 5844 |
| J04.0 | Acute laryngitis | | 2491 |
| J06.0 | Acute laryngopharyngitis | | 1632 |
| J06.8 | Other acute upper respiratory infections of multiple sites | | 1372 |
| J04.2 | Acute laryngotracheitis | | 1053 |
| J01.1 | Acute frontal sinusitis, unspecified | | 1020 |
| J01.9 | Acute sinusitis, unspecified | | 962 |
